# Immunologic biomarkers, morbidity and mortality among HIV patients hospitalised in a Tertiary Care Hospital in the Brazilian Amazon

**DOI:** 10.1186/s12879-021-06566-x

**Published:** 2021-08-26

**Authors:** Wellington Mota Gama, Carlos Henrique Michiles Frank, Taynná Vernalha Rocha Almeida, Daniel Silva dos Santos, Yury Oliveira Chaves, Danielle Furtado da Silva, Patrícia Puccinelli Orlandi, Flávio Ribeiro Pereira, Gleicienne Feliz Magalhães, Bárbara Jóse Baptista, Viviane Lago de Oliveira Silva, Antônio Alcirley da Silva Balieiro, Monique Freire Santana, Roberta Lins Gonçalves, Allyson Guimarães da Costa, Marcelo Cordeiro dos Santos, Luís Carlos de Lima Ferreira, Marcus Vinicius Guimaraes Lacerda, Paulo Afonso Nogueira

**Affiliations:** 1 Programa de Pós-Graduação em Imunologia Básica e Aplicada, Universidade Federal do Amazonas, Manaus, Brazil; 2 Fundação de Medicina Tropical Dr. Heitor Vieira Dourado, Manaus, Brazil; 3 Programa de Pós-Graduação em Biologia da Relação Patógeno Hospedeiro, Instituto Leônidas e Maria Deane, Manaus, Brazil; 4 Programa de Pós-Graduação em Biologia Parasitária, Instituto Oswaldo Cruz, Rio de Janeiro, Brazil; 5 Programa de Pós-Graduação em Ciências da Saúde, Universidade Federal do Amazonas, Manaus, Brazil; 6 Instituto Aggeu Magalhaes, Fundação Oswaldo Cruz-Fiocruz, Recife, Brazil; 7 Programa de Pós-Graduação em Medicina Tropical, Universidade do Estado do Amazonas, Manaus, Brazil; 8 Diretoria de Ensino e Pesquisa, Fundação Hospitalar de Hematologia e Hemoterapia do Amazonas, Manaus, Brazil; 9 Programa de Pós-Graduação em Ciências Aplicadas à Hematologia, Universidade do Estado do Amazonas, Manaus, Brazil

**Keywords:** (MeSH):HIV, Acquired immunodeficiency syndrome, AIDS-related opportunistic infections, Biomarkers, Morbidity, Mortality

## Abstract

**Background:**

The irregular use of antiretroviral therapy (ART) and late diagnosis still account for a large part of HIV-associated mortality in people living with HIV (PLHIV). Herein, we describe HIV-associated morbidity among hospitalised HIV/AIDS patients with advanced immunosuppression and assess the comorbidities, laboratory parameters, and immunological markers associated with mortality.

**Methods:**

The cross-sectional study was conducted at the Fundação de Medicina Tropical Doutor Heitor Vieira Dourado (FMT-HVD) in Manaus, Brazil. In all, 83 participants aged between 12 and 70 years were enrolled by convenience within 72 h of their hospitalisation. Clinical and laboratory data were obtained from electronic medical records. We prospectively measured the cytokines Th1/Th2/Th17 and inflammatory cytokines IL-8, IL-1β, and IL-12 using cytometric bead array, and the soluble CD14 using in-house enzyme-linked immunosorbent assay.

**Results:**

The HIV/AIDS inpatients presented a scenario of respiratory syndromes as the most prevalent comorbidity. Almost all patients had CD4 T counts below 350 cells/mL and the mortality rate was 20.5%. Pulmonary tuberculosis, neurotoxoplasmosis and oropharyngeal–esophageal candidiasis were the most prevalent opportunistic infections. TB and weight loss were more prevalent in HIV/AIDS inpatients who died. The Mann Whitney analysis showed that those who died had higher platelet distribution width (PDW) on admission, which is suggestive for platelet activation. The Poisson multivariate analysis showed the prevalence of TB, digestive syndrome and increases in IL-8 and lactate dehydrogenase (LDH) associated to death.

**Conclusions:**

The advanced immunosuppression characterized by the opportunistic infections presented in these HIV/AIDS inpatients was the major factor of mortality. The role of platelet activation in worse outcomes of hospitalisation and the IL-8 associated with the context of advanced immunosuppression may be promising markers in the prediction of mortality in HIV/AIDS patients.

## Background

Brazil has been successfully combating the HIV/AIDS epidemic by providing antiretroviral therapy (ART) in a unified, universal and free public health system that is widely available to the population [1, 2]. The mortality rate due to HIV/AIDS in Brazil has fallen significantly during the last 20 years, especially in the southeastern, southern and mid-western regions. This has been principally influenced by a government policy of linking HIV/AIDS treatment and medication dispensing to compulsory notification within the public health system (Sistema Único de Saúde/SUS) [1, 3, 4]. This system has been fundamental for the decision-making process at the government level in regard to providing access to ART for all PLHIV.

Since the beginning of the epidemic in 1982, more than 300,000 deaths caused by HIV/AIDS have been reported in Brazil. After the adoption of ART in 1996, there was a significant reversal in mortality. The deaths related to the HIV/AIDS epidemic have shown a downward trend in Brazil as a whole, however this trend is still subtle in the northern and northeastern regions [1, 3, 5]. Similarly, the number of new cases of HIV/AIDS has been gradually decreasing in recent years, influenced by the southeast, southern and central-western regions, while the northern and northeastern regions still show a growth in new cases [3, 4].

Located in northern Brazil, the State of Amazonas has peculiar characteristics, such as geographic isolation and, in the interior of the state, a lack of access to full health services. The capital, Manaus, is the largest city in the state and its health units are decentralized, located in the neighborhoods and semi-urban areas. These health units are essential for promoting the early diagnosis and the introduction of ART, and contribute to the decrease in morbidity and mortality caused by opportunistic diseases [2]. The Fundação de Medicina Tropical Doutor Heitor Vieira Dourado (FMT-HVD) is a tertiary care hospital, which provides inpatient and outpatient care for PLHIV. The FMT-HVD is responsible for more than 85% of all patients receiving ART, and also concentrates vital data on incidence levels of AIDS, mortality rates and late diagnosis [2, 3, 5–8]. We still see many cases of virological failure and late presentation (e.g., patients presenting a late diagnosis or patients that have been infected for longer periods before being diagnosed) [2, 5, 8]. Recently, we observed in a cross-sectional study with older than 18 years of age underwent antiretroviral therapy at least 6 months [8]. This reflects in deaths related to the HIV/AIDS, especially among PLHIV from black/brown/indigenous race/color or those having lower levels of education. As they have less access to ART or have been in discontinuous ART, the consequence is no virological suppression [6, 9]. Studies on the HIV/AIDS epidemic in the Amazon region have indicated that over time the trend in the number of cases has continued to increase, principally in men. Concerning PLHIV, TB stands out as the main cause of death and respiratory failure as the main cause of hospital admission [5, 10–12].

Immune activation in HIV infection is a strong predictor of disease progression and is a result of dysregulated innate and adaptive immune responses to HIV with active participation of co-infections and microbial products [13, 14]. In PLHIV who are ART naïve or among those who have interrupted their ART, the risk of dying is higher than those who were treated continuously [15, 16]. Thus, the need to evaluate factors associated with the risk of death is increased in order to ensure an appropriate investigation and treatment during the hospitalisation of these vulnerable people. In this study, we report the prevalence of coinfections and comorbidities and assess laboratory and immunological markers within 72 h of hospital admission associated with the risk of death in HIV/AIDS inpatients.

## Methods

### Study population and study design

This cross-sectional study assessed the prevalence of comorbidities, laboratory parameters, and the immunological markers associated with the death in HIV/AIDS patients of either sex, who were admitted to the FMT-HVD between 2017 and 2018. Eighty-three participants aged between 12 and 70 were enrolled in this study by convenience within 72 h of admission. After signature of the informed consent form, the patient’s blood was collected and the serum cytokines, chemokines, and soluble CD14 were measured.

### Clinical data

During the first contact with the patients, socio-demographic data, such as name, age, gender, and use of ART, were collected. The following data were collected through the patient’s electronic medical records:

- Clinic data: general health status, comorbidities, co-infections, treatment, clinical manifestations (weight loss, diarrhea, vomiting) and death.
- Laboratory data: blood count, the IgG serology test (cytomegalovirus—CMV, toxoplasmosis, Epstein Barr—EBV, herpes virus, hepatitis B virus—HBV, hepatitis C virus—HCV), immunological markers (viral load and T cell count CD4+ T and CD8+ T) and biochemical markers (bilirubin, creatinine, lactic dehydrogenase, GT range, albumin, alkaline phosphatase, GPT and OGT).

### General characteristics of patient comorbidities

Information regarding coinfections and comorbidities were obtained from the electronic medical database at the FMT-HVD, and the outcomes of interest were survival (hospital discharge) and death. Both were verified via either a death certificate or discharge authorization registered on the electronic medical record. Comorbidities or disorders were defined as signs and/or symptoms of respiratory, neurological, cardiovascular, and digestive origin, of infectious and non-infectious cause, with or without chronicity.

Respiratory disorders encompass a variety of pathogenic conditions that affect the respiratory tract, and include infectious and non-infectious signs and symptoms such as dyspnea (shortness of breath or difficulty in breathing), abnormal lung auscultation, long-term and/or productive cough, and pleural effusion. Neurological disorders include those of infectious and non-infectious etiology, signs and symptoms such as alteration in consciousness, sensory loss and/or movement disorders (poor coordination, tremors, asthenia), paralysis, and seizure. The cardiovascular disorders included hypertension, heart failure, peripheral edema, and anasarca. Gastrointestinal disorders include those of an infectious and non-infectious cause, signs and symptoms such as include odynophagia, dysphagia, esophagitis, gastritis, vomiting, and diarrhea.

Other comorbidities, such as chronic lymphocytic leukemia, Hodgkin’s disease, aplastic anemia, and neurological disorders, such as multiple sclerosis and myasthenia gravis, were monitored.

### Blood sample collection

On the same day as the patient’s enrollment, after the interview and signing of the consent form, 5 mL of blood was collected by vacuum venipuncture. The samples were collected in dry tubes and centrifuged at 3500 rpm for 5 min at 25 °C to obtain the serum and aliquoted (1 mL) for analysis of the inflammatory markers. The whole blood that was collected in an anticoagulant tube was homogenized, aliquoted (1 mL), and then stored for future analysis. Samples were stored at − 80 °C until use.

### Immunological markers

The measurement of serum cytokines was performed using the flow cytometry technique CBA (cytometric bead array) with the human cocktail Th1/Th2/Th17 cytokine for IL-2, IL-4, IL-6, IL-10, TNF-α, IFN-γ and IL17 and inflammatory cytokines IL-8, IL-1β, and IL-12 (Biosciences, USA). Serum concentrations of soluble CD14 (sCD14) were determined by *in-house* enzyme-linked immunosorbent assay (ELISA). Initially, the plate was sensitized with the primary antibody (anti-human CD14 Antibody; Cat. Nº. MAB3833; Lot. AWI091610A; R&D Systems) diluted in carbonate–bicarbonate buffer (0.05 M; pH 9.6). After sensitization, blocking was performed with PBS/BSA blocking buffer (PBS pH 7.4; 3% bovine albumin). Subsequently, the sera from patients and controls were diluted into PBS/BSA dilution buffer (PBS pH 7.4; 0.5% bovine albumin) and incubated for 1 h at 37 °C. Three concentrations of recombinant human CD14 protein (Recombinant Human CD14; Cat. No. 383-CD; Lot. BCS1716091; R&D Systems) were added to each plate well in order to determine a standard-curve. After four lavage steps in PBS washing buffer (PBS pH 7.4; 0.05% Tween 20), the 1:1000 dilution secondary antibody (anti-human CD14 Biotinylated Antibody; Cat Nº BAF383; Lot. BAR0714031; R&D Systems) was added. After the lavage steps, 1:1000 dilution, 0.5 mg, peroxidase-labeled streptavidin (KPL) was added for 1 h at 37 °C. The reaction was developed with developing buffer (citrate-phosphate buffer + TMB + H_2_O_2_ 30%) and stopped with H_2_SO_4_ (2.0 M). The reading was made by colorimetric means (optical density—OD), using a spectrophotometer for an ELISA plate reader (Bio-Rad iMark™ microplate reader) at an absorbance of 450 nm.

### Statistical analysis

Data were tabulated in an Excel database created by the researchers and analyzed using the GraphPad Prism program, version 7. For data analysis, patients were defined as Death (those who died during hospitalisation) or Discharge (those who were discharged from hospital). Descriptive analysis was performed with mean and standard deviation (SD) for numerical variables when distribution was normal, or median and interquartile range for non-normal distributions. In the univariate analysis, Fisher’s exact test was used to determine relative risk among categorical variables. Laboratory parameters, soluble CD14, cytokine and chemokines concentrations, and days of hospitalisation were compared between the Death and Discharge groups using the Mann Whitney test. The Poisson regression was performed to assess factors associated with death in HIV/AIDS inpatients among the most prevalent comorbidities, laboratory parameters and immunological markers. For this model, we used data from univariate analyses where the selected variables were those presenting p < 0.2. The statistical significance was defined as p < 0.05.

## Results

Overall, respiratory disorders were the most prevalent (62.1%), followed by neurological (37.9%), gastrointestinal (21.0%) and cardiovascular (5.2%) disorders. Among the respiratory disorders of infectious cause, TB was the most prevalent (56.30%), followed by pneumocystosis (2.4%). The most prevalent signs and symptoms were abnormal lung auscultation (22.40%), followed by respiratory failure (5.30%), and severe cough (5.30%). Regarding neurological disorders, the most prevalent coinfections were neurotoxoplasmosis (44.4%), neurocriptococcosis (8.3%), and neurotuberculosis (2.8%). The most prevalent signs and symptoms were confusion, paralysis, and/or poor coordination (30.6%), tremors and asthenia (11.1%), and seizures (5.6%). The most prevalent gastrointestinal disorders of infectious cause were oral and esophageal candidiasis (55%). The most prevalent signs and symptoms were vomiting (39.7%), diarrhea (33.7%), and odynophagia (5.0%). A total of 25% of patients reported abdominal pain.

In all, 17 hospitalised patients died and 66 were discharged. The duration of hospitalisation did not differ between the two groups (p = 0.30), the median and 25% and 75% interquartiles of hospitalised days of those who died were 19 (14.5–26.5), while for those who were discharged the median was 25 (11.0–38.0). All the described disorders were assessed to identify factors associated with the mortality of the patients (Table 1). Tuberculosis was more prevalent among patients who died (p = 0.001). The IgG serological tests used for previous infections did not differ between HIV/AIDS patients who died or were discharged from hospital. Weight loss was associated with death (*p* = 0.013). The univariate analyses of HIV-RNA > 1000 copies and CD4 T < 350 cells/mL showed no association with death. The CD4 T counts were greatly reduced in both groups, the CD8 T cells presented normal values in the group of patients who died, while the Discharge group tended towards a slight increase, though this was not statistically significant. The ratio of CD4:CD8 cells below 0.20 due the reduction of CD4-T evidenced the advanced immune impairment of the patients (Table 2).

**Table 1:** Assessment of comorbidities in the outcomes of HIV–AIDS inpatients

Clinical data	Death	Discharge	*p*
	N = 17	N = 66	
Age (mean, std.)	37.6 (9.9)	36.2 (9.4)	0.590
Gender (male)	11 (64.7)	54 (81.8)	0.184
ART	12 (70.6)	55 (83.3)	0.300
CD4 T < 350 cells/mL	14 (82.4)	58 (87.9)	0.688
HIV-RNA > 1000 copies	14 (82.4)	47 (71.2)	0.539
Weight loss	14 (82.4)	30 (45.5)	0.007**
Very good condition	7 (41.2)	35 (53.0)	0.425
Lucid and space-oriented	8 (47.1)	40 (60.6)	0.410
Anemia	15 (88.2)	43 (65.2)	0.079
Respiratory disorder	14 (82.4)	42 (63.6)	0.244
Neurological disorder	8 (47.1)	29 (43.9)	0.999
Cardiovascular disorder	2 (11.8)	2 (3.0)	0.184
Gastrointestinal disorder	8 (47.1)	16 (24.2)	0.077
Active coinfections			
Tuberculosis	12 (70.6)	25 (37.9)	0.026*
Parasites in stools	3 (17.6)	13 (19.7)	0.999
Toxoplasmosis IgM	1 (5.9)	7 (10.6)	0.999
Cytomegalovirus IgM	1 (5.9)	2 (3.0)	0.502
Epstein Barr virus IgM	17 (100)	66 (100)	0.999
Herpes virus IgM	0 (0)	3 (4.5)	0.999
Previous infections			
HCV IgG	1 (5.9)	2 (3.0)	0.502
Cytomegalovirus IgG	13 (76.5)	53 (80.3)	0.741
Toxoplasmosis IgG	13 (76.5)	52 (78.8)	0.999
Epstein Barr virus IgG	12 (70.6)	49 (74.2)	0.764
Herpes virus IgG	12 (70.6)	44 (66.7)	0.999

*, **: *p*-value; statistically significant difference

**Table 2:** Univariate analyses with laboratory parameters in the outcomes in HIV/AIDS patients

Laboratory tests	Death	Discharge	*p*
	N = 17	N = 66	
Hematological data (NVA)			
HIV-RNA copies/mL^b^	126,578 (3247–273,012)	25,258.5 (391.5–145,976.8)	0.590
Lymphocytes (percent)^a^ (25.0–40.0%)	20.5 (10.3)	27.4 (13.5)	0.055
CD4-T cells/µL^b^ (600 to 1500/mm^3^)	44 (7–232)	79 (30–198.2)	0.140
CD8-T cells/µL^b^ (200 to 800/mm^3^)	402 (198–776)	544.5 (359.2–1143.8)	0.312
CD4/CD8 ratio^b^ (1.0 and 4.0)	0.1 (0–0.4)	0.1 (0.1–0.3)	0.123
Hemoglobin (13.0–18.0 g/dL)	9.3 (2.7)	10.8 (2.4)	0.165
Leukocytes^b^ (4500–11,000/mL)	4740 (3510–8600)	4215 (3275–5,757.5)	0.527
Neutrophils^b^ (1800–7700/mL)	2660 (1679–3234)	3118 (2109–4907.5)	0.113
Monocytes^b^ (80–1100/mL)	249 (161–379)	329 (187.2–488.2)	0.370
Eosinophils^b^ (40–550/mL)	2 (2–4)	4 (2.6–7)	0.030*
Platelets^a^ (150,000–400,000 × 10^3^/mL)	299,352.9 (207,630.4)	273,947.0 (132,454.6)	0.536
MPV^b^ (8.8 fL to 12.5 fL)	8.2 (7.8–8.8)	8.1 (7.4–8.7)	0.446
PDW^b^ (9.3 fL to 16.0 fL)	15.8 (13–18.7)	13.5 (11.2–16.5)	0.037*
Biochemistry parameters (NVA)			
Bilirubin^b^ (< 1.0 mg/dL)	0.4 (0.2–0.9)	0.4 (0.3–0.7)	0.999
Creatinine^b^ (0.6–1.35 mg/dL)	1 (0.7–1.3)	0.8 (0.6–0.9)	0.069
LDH^b^ (120 and 246 U/uL)	435 (332–534)	367.5 (300.2–474.2)	0.159
Gamma-glutamyltransferase^b,c^	88 (42–203)	103.5 (57.8–290)	0.876
Albumin^b^ (3.5–5.0 g/dL)	3.4 (2.8–4)	3.8 (3.3–4.5)	0.129
Alkaline phosphatase^b^ (65.0–330.0 U/uL)	282 (200–459)	281 (201–386)	0.835
Aspartate aminotransferase^b^ (2–38.0 U/uL)	44 (26–82)	34 (25–58)	0.280
Alanine aminotransferase^b^ (2–44.0 U/uL)	31 (22–41)	43 (31–77)	0.038*

*NVA* Normal values in adults, *PDW* Platelet distribution width, *MPV* Mean platelet volume, *LDH* lactate dehydrogenase

^a^Mean and standard

^b^Median (25th; 75th interquartile)

^c^References values of Gamma-glutamyltransferase (men: 10 to 50 U/uL and women: 7 to 32 U/uL)

*, **: *p*-value; statistically significant difference

The low hemoglobin level was present in both groups; however, it was more prevalent in those who died. The total leukocytes, neutrophils, and monocytes did not differ between groups and remained in normal ranges in most patients. The percentage of lymphocytes in those who died showed a tendency to be lower. The eosinophil counts were reduced in both groups, though they were even lower (p = 0.03) in those who died. The platelet distribution width (PDW) was higher among those who died.

Overall, the patients preserved their liver function, with no significant alterations in the hepatic markers. The alkaline phosphatase levels and the transaminase levels were slightly elevated in both groups. The renal function was able to maintain the creatinine levels in normal ranges (Table 2).

**Fig. 1:**
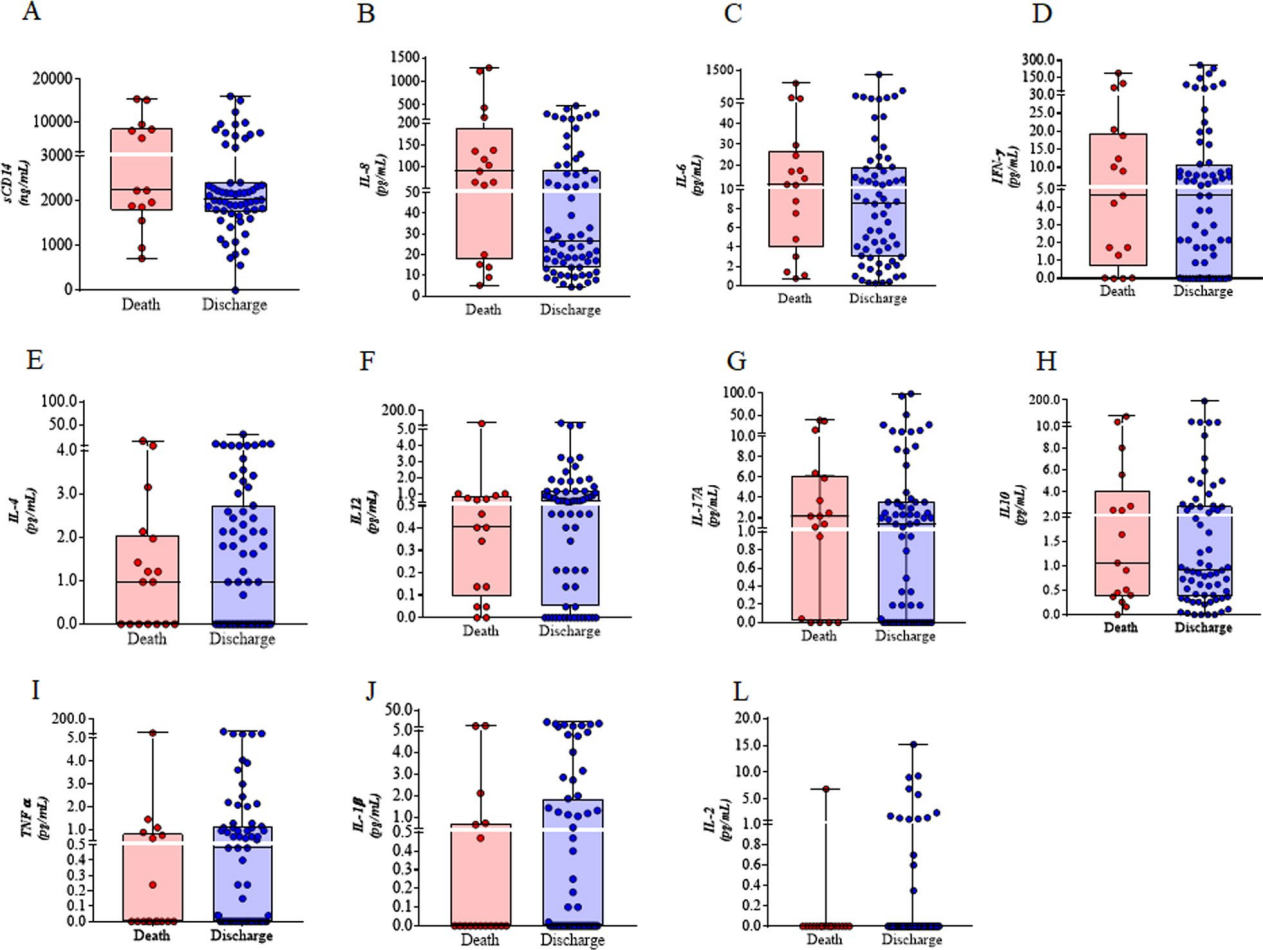
Serological markers as predictors of mortality. The soluble CD14, the chemokine IL-8 and the cytokines Th1/Th2/Th17 were compared among the patients who died and those who survived. **A** sCD14; **B** IL-8; **C** IL-6; **D** IFNγ; **E** IL-12; **F** IL-2; **G** IL-4; **H** IL-17A; **I** IL-10; **J** TNFα and **L** IL-1β. Concentrations were compared between Death and Survival groups using the Mann Whitney test

The soluble CD14, the chemokines and Th1/Th2/Th17 cytokine levels at the time of hospitalisation revealed no difference between the two groups, while IL-8 tended to be associated with death (p = 0.06) (Fig. 1). It is worth mentioning the stratification of immunological markers in descending order of plasma levels.

When isolated, the prevalence of TB showed a relative risk (RR) of 4.62, though, when associated with digestive syndrome, it presented RR = 3.07. In relation to laboratory parameters and immunological markers, the risk of death increases along with the increment in IL-8 and LDH levels, e.g., IL-8 (RR = 1.0001) and LDH (RR = 1.0007). The mean IL-8 in univariate analysis showed no differences between groups; however, most patients who died had higher levels of IL-8 than those who were discharged (Fig. 1B). The RR of the lymphocyte percentage was 0.9503, indicating that the risk of death was higher as the percentage decreased (Table 3).

**Table 3:** Relative risk of comorbidities, laboratory parameters and immunological markers in the mortality of HIV/AIDS patients

Characteristic	Death		RR	95% CI	p-value
	Yes	No			
	N = 17	N = 66			
Digestive syndrome^a^	8/17 (47%)	16/66 (24%)	3.05	(1.55–6.06)	0.001
Tuberculosis^a^	12/17 (71%)	25/66 (38%)	4.62	(1.87–11.36)	0.001
IL.8 pg/mL^b^	90.4 (20–189.0)	26.1 (14.0–90.1)	1.01	(1.00–1.01)	0.007
Lymphocytes^b^ in percentage	20.5 (10.3)	27.4 (13.5)	0.95	(0.91–0.98)	0.003
LDH U/uL** (120 and 246 U/uL)	435 (332–534)	367.5 (300.2–474.2)	1.01	(1.00–1.01)	0.001

Relative risk; CI: confidence interval; and the p-value was calculated using the Poisson regression; LDH: lactate dehydrogenase

^a^Among the categorical variables, the statistics were presented as n/N (%); where n = number of patients for whom the variable was positive. N represents total number in each group

^b^Among the numerical variables, statistics were presented as median (Q1–Q3) or mean (SD)

**: References value of lactate dehydrogenase (120 and 246 U/uL)

## Discussion

After the advent of ART, HIV infection has changed from an illness that causes rapid deterioration to a complex chronic disease. Brazil has responded to the HIV pandemic in innovative ways, and was the world’s first developing country to offer ART free of charge to all PLHIV and the third developing country to provide ART regardless of CD4+ T cell counts [1, 17]. As a consequence, the HIV/AIDS mortality rate continues to fall [3]. However, the states of Acre, Amazonas, and Roraima in northern Brazil still show increases in mortality. PLHIV with poorer outcomes in these states have a strong association with both late diagnosis and their consequent late assistance, and virologic failure (non-achievement or non-maintenance of undetectable viral load). The sociodemographic determinants, such as poverty, race/color (black/brown/indigenous), schooling, and social vulnerability index are still an obstacle for accessing timely diagnosis and proper treatment [3, 6–8].

This cross-sectional study has found that the HIV/AIDS epidemiological scenario has not changed in the few last years; respiratory disorders are still the most prevalent complication, while TB, neurotoxoplasmosis, and oropharyngeal–esophageal candidiasis are the most prevalent opportunistic infections. During the last decade, TB has been highly prevalent among HIV/AIDS patients who died [5, 10, 11, 18]. Additionally, we found weight loss associated to death through the univariate analysis, in agreement with early mortality in TB-HIV coinfected patients presenting malnutrition or low body mass index [19, 20]. It is worth mentioning that weight loss and gastrointestinal disorders can be correlated variables [20]. Our multivariate analysis corroborated greater risk of death among those TB-HIV coinfected patients, similar with those coming from low-income regions, whose poverty, schooling, and social vulnerability index decrease the odds of access ART [6, 19, 21–26]. We observed an augmented risk of death among those patients presenting gastrointestinal disorders, especially oropharyngeal–esophageal candidiasis, which is recognized as an indicator of advanced immunosuppression [27]. This fungal infection is helping physicians to diagnose the clinical progression and risk of death [27, 28].

Another challenge is the patients’ low adherence to ART. Despite the free access to the antiretroviral drugs, due to several reasons including social factors, side effects or skepticism, since many have experienced previous treatment failure, some patients abandon ART [29]. In the latter cases, we observed both high prevalence of individuals with virologic failure without viral drug resistance mutations (DRMs) and an increment of transmitted HIV-DRMs, which has contributed to one of the worst epidemiological scenarios in northern Brazil in the last few years [8].

As almost all HIV-infected patients from both groups showed the CD4 counts lower than 200 cells/uL. However, the risk of death associated with decreased CD4 was especially a result of TB prevalence associated with oropharyngeal–esophageal candidiasis. The univariate analysis showed higher PDW at the time of admission of the patients who died. PDW is a platelet activation marker, since, under activation, platelets increase in number and size of pseudopodia, affecting their distribution width [30]. Platelets are small, anucleate cells that have hemostatic and inflammatory proprieties, and are considered more than just innocent bystanders. Our findings point towards the platelet activation being associated with severity of the systemic inflammatory responses in TB and bacterial sepsis [31–33].

HIV/AIDS is characterized by a state of chronic immune activation and inflammation. Elevated levels of IL8 have been reported in plasma, serum and cerebrospinal fluid in HIV/AIDS patients with CD4+ T cell counts < 350 cells/mm^3^, a promising marker of the inflammatory process [34–36]. Our multivariate analysis evidenced the association of digestive disorder, TB, elevated levels of IL-8 and LDH, and low levels of CD4 with increased risk of death [37–40].

IL-8 is a prominent neutrophil chemoattractant produced by macrophages and plays a dual role in the pathogenesis of TB and HIV-1 coinfection. On the one hand, IL-8 performs a key role in killing *M. tuberculosis* by phagocytosis in in vitro studies [41, 42]. On the other hand, it enhances HIV-1 replication in monocyte derived macrophages and T lymphocytes [41, 43]. An important mechanism of host defense against TB is the granuloma. IL-8 has a significant role since it regulates the leukocyte influx in its formation. One in vivo study demonstrated that pretreatment with anti-IL-8 alone inhibits mycobacterial granuloma formation [44, 45]. Moreover, IL-8 is a major inflammation-associated cytokine in pneumonia, and is correlated with neutrophil infiltration of the lung and impairment of the gas exchange [37]. In this context, an elevated IL-8 level was considered to be a factor associated with higher mortality in HIV/AIDS patients with opportunist infections, especially those precipitating respiratory disorders such as bacterial, viral and fungal pneumonia [35–40, 46, 47]. Therefore, our findings are in agreement with studies that show that IL-8 may be used as a serological predictor for death in a scenario of advanced HIV/AIDS with TB and fungal coinfections [46, 48–52].

The sample size was one limitation of this study, and restricted the detection of other associations. Several factors contributed to the limitation of the sample, one of which is that the study was carried out in a medium-sized hospital. In addition to the structural limitations, some patients were hospitalised for up to 2 months, and others were re-hospitalised. Nevertheless, our limited sample size (by convenience) is still representative of the HIV/AIDS population in the region. Before ART, Kaposi’s sarcoma and pneumocystosis were the most prevalent opportunistic diseases; after ART, TB, bacterial pneumonia and histoplasmosis became the most frequent causes of death. This study sampling was able to represent the same epidemiological scenario regarding HIV/AIDS patients that has been prevalent in northern Brazil in recent years.

Another limitation was related to the study design, which did not include patients’ follow up, only one single blood sample collection. The information regarding the deaths of patients was obtained solely via electronic medical records.

## Conclusions

This cross-sectional study found that the HIV/AIDS epidemiological scenario remains unchanged in northern Brazil, e.g., greater risk of death among the TB-HIV coinfected patients and among those with advanced immunosuppression, characterized by the presence of oropharyngeal–esophageal candidiasis. The univariate analysis showed higher PDW on hospital admission, indicating platelet activation, though further studies are needed to assess their relationship with the severity of the pulmonary infection in HIV/AIDS patients. Nonetheless, the use of IL-8 as a serological predictor of death in the HIV/AIDS patients is promising.

## Data Availability

The datasets used and/or analyzed during the current study are available from the corresponding author on reasonable request.

## Abbreviations

AIDS: Acquired immunodeficiency syndrome
ART: Antiretroviral therapy
CBA: Cytometric bead array
CI: Confidence interval
CMV: Cytomegalovirus
EBV: Epstein Barr virus
FMT-HVD: Fundação de Medicina Tropical Dr. Heitor Vieira Dourado
HBV: Hepatitis B virus
HCV: Hepatitis C virus
HIV: Human immunodeficiency virus
IFN: Interferon
IL: Interleukin
MPV: Mean platelet volume
NVA: Normal values in adults
PDW: Platelet distribution width
RR: Relative risk
TB: Tuberculosis
TNF: Tumor necrosis factor
